# Procedure code overutilization detection from healthcare claims using unsupervised deep learning methods

**DOI:** 10.1186/s12911-023-02268-3

**Published:** 2023-09-28

**Authors:** Michael Suesserman, Samantha Gorny, Daniel Lasaga, John Helms, Dan Olson, Edward Bowen, Sanmitra Bhattacharya

**Affiliations:** 1 AI Center of Excellence, Deloitte & Touche LLP, New York, NY USA; 2 Program Integrity, Deloitte & Touche LLP, New York, NY USA

**Keywords:** Fraud, waste, and abuse, Procedure code overutilization, Unsupervised learning, Deep autoencoder, Feature-weighted loss function

## Abstract

**Background:**

Fraud, Waste, and Abuse (FWA) in medical claims have a negative impact on the quality and cost of healthcare. A major component of FWA in claims is procedure code overutilization, where one or more prescribed procedures may not be relevant to a given diagnosis and patient profile, resulting in unnecessary and unwarranted treatments and medical payments. This study aims to identify such unwarranted procedures from millions of healthcare claims. In the absence of labeled examples of unwarranted procedures, the study focused on the application of unsupervised machine learning techniques.

**Methods:**

Experiments were conducted with deep autoencoders to find claims containing anomalous procedure codes indicative of FWA, and were compared against a baseline density-based clustering model. Diagnoses, procedures, and demographic data associated with healthcare claims were used as features for the models. A dataset of one hundred thousand claims sampled from a larger claims database is used to initially train and tune the models, followed by experimentations on a dataset with thirty-three million claims. Experimental results show that the autoencoder model, when trained with a novel feature-weighted loss function, outperforms the density-based clustering approach in finding potential outlier procedure codes.

**Results:**

Given the unsupervised nature of our experiments, model performance was evaluated using a synthetic outlier test dataset, and a manually annotated outlier test dataset. Precision, recall and F1-scores on the synthetic outlier test dataset for the autoencoder model trained on one hundred thousand claims were 0.87, 1.0 and 0.93, respectively, while the results for these metrics on the manually annotated outlier test dataset were 0.36, 0.86 and 0.51, respectively. The model performance on the manually annotated outlier test dataset improved further when trained on the larger thirty-three million claims dataset with precision, recall and F1-scores of 0.48, 0.90 and 0.63, respectively.

**Conclusions:**

This study demonstrates the feasibility of leveraging unsupervised, deep-learning methods to identify potential procedure overutilization from healthcare claims.

**Supplementary Information:**

The online version contains supplementary material available at 10.1186/s12911-023-02268-3.

## Background

Each year billions of insurance claims are submitted by healthcare providers. In 2019, the U.S. healthcare spending grew 4.6 percent to $3.8 trillion, which was 17.7 percent of the Gross Domestic Product [1], and healthcare costs are projected to grow to over $6 trillion by 2028 [2]. However, fraud, waste, and abuse (FWA) in healthcare claims pose a significant risk to patient care and accessibility to health services. The National Health Care Anti-Fraud Association conservatively estimates healthcare fraud at 3 percent of total healthcare costs, which in 2019 represented over $100 billion in fraud, and some estimates of healthcare fraud go as high as 10 percent, which represents almost $400 billion in fraud [3, 4].

Various forms of FWA may be observed in healthcare claims. For example, kickbacks [5] are a type of fraud where there is a collusion between a patient and a provider to gain commission for services that are not rendered or illegal. Upcoding is another type of FWA where a provider submits inaccurate and expensive billing codes which would result in inflated reimbursements. Bauder et al. [6] present a survey of 26 papers on machine learning approaches to detect upcoding from healthcare claims data. They find across these papers various supervised, unsupervised, and hybrid learning methods applied to healthcare claims from governmental health departments and private insurers to identify upcoding fraud. They also highlight some major challenges in current approaches, such as reliance on high-quality labeled data for supervised models, and inability of static models to capture the dynamic nature of fraudulent behaviors. Joudaki et al. [7] presents an overview of various data mining approaches to identify provider and patient fraud. They recommend extensive feature engineering techniques for data preparation, application of supervised methods for online processing tasks for known patterns of fraud, and unsupervised approaches at specific time periods for detecting new fraud patterns. While several other studies [8–10] have explored machine learning approaches for different types of fraud and anomaly detection in healthcare claims, we focus on a relatively unexplored FWA problem – procedure code overutilization detection.

Procedure code overutilization is a type of healthcare FWA where a healthcare provider submits a claim with inappropriate or unnecessary procedure codes [11] (in the form of Current Procedural Terminology (CPT) or Healthcare Common Procedure Coding System (HCPCS)) [12]. Overutilization is the largest component of waste in the U.S. healthcare system [13]. Overuse is estimated to represent between 20 and 30 percent of total healthcare costs [14–16], which would have been about $1 trillion in 2019. Identifying instances of overutilization is an important part of making sure that patients get the most appropriate care at the lowest possible cost.

Traditionally, state Medicare and healthcare agencies have relied on rules-based and volume-based analysis using a Surveillance Utilization Review System (SURS) to identify and reduce potential overutilization [17]. Although required by federal regulation, each state decides how to design their SURS. In general, it simultaneously analyzes multiple claims as part of identifying billing patterns that potentially indicate overutilization, which could then be further reviewed by healthcare regulators. Lack of standardization means that SURS’ performance varies widely based on the specific implementation in each state. Prior research on overutilization detection using machine learning approaches is quite limited, with Lasaga and Santhana [18] demonstrating the application of Restricted Boltzmann Machines (RBM) for outlier treatment detection. Training and evaluation of RBM was performed on a simulated dataset with only 800 treatment and 400 diagnosis codes, with 10% simulated fraud injected into the dataset. In contrast, in this paper we propose unsupervised machine learning approaches that learn to directly detect procedure code overutilization from Medicare claims containing over 6700 diagnosis and procedure codes. To achieve this, a density-based clustering model and an autoencoder model are trained on large historical claims databases containing information on diagnosis codes, procedure codes, and patient demographics. In our experiments the autoencoder model outperforms the density-based model by learning feature representations that capture the key regularities in the data to minimize reconstruction errors, while resulting in larger reconstruction errors for outlier procedures.

Our key contributions in this paper are: 1) to the extent of our knowledge, this is the first paper to introduce a practical approach for addressing the procedure code overutilization detection problem with unsupervised machine learning models applied to Medicare claims data, and 2) we implement a novel feature-weighted loss function for the autoencoder model that guides the model towards identifying outlier procedures in a highly imbalanced dataset with sparse feature representations.

## Methods

In this section, we discuss details of the healthcare claims data, pre-processing and feature representation, and details of the modeling approaches. The models were validated using a synthesized out-of-sample outlier dataset, and a labeled dataset consisting of manually annotated healthcare claims scored by FWA subject matter specialists (SMS) as to their likelihood of containing procedure overutilization. We discuss the methodology of creating these two test datasets.

### Data overview and pre-processing

We used two datasets in this study – one with one hundred thousand claims (referred to as 100k_claims dataset hereafter) and another one with thirty-three million claims (referred to as 33M_claims dataset hereafter). Both datasets used in this study comprise of anonymized and redacted outpatient medical claims from state Medicare programs. An outpatient claim refers to one where a patient visits a healthcare provider but does not get admitted to a hospital. The 100k_claims dataset was used for benchmarking model performance and model selection, while the 33M_claims dataset was used to train our final model for production. Both datasets share the same features which are discussed below:

- Claim Number: an identifier for each claim.
- Diagnosis Codes: represented by International Classification of Diseases (ICD-10) codes [19] that specify the codified medical diagnosis for a patient as submitted by a healthcare provider.
- Procedure Codes: represented by Current Procedural Terminology (CPT) or Healthcare Common Procedure Coding System (HCPCS) codes [12, 20, 21] that indicate the codified procedures performed for the given diagnosis, as submitted by a healthcare provider.
- Patient Demographics: age at the time of claim submission (derived from the difference between date of submission of the claim and patient’s date of birth), and gender.
- Provider ID: an identifier for the healthcare provider in the form of a National Provider Identifier (NPI) [22].
- Member ID: an identifier for the Medicare beneficiary/patient.
- Claim Start and End Dates: the dates the first and last procedures associated with a claim are performed.
- Billed Amount: how much the healthcare provider billed for the procedures.

A healthcare provider gets paid based on the specific procedure codes included in a claim. For this study, we use diagnosis codes, procedure codes, patient age, and gender associated with a claim as features for the models.

Besides the features stated above, the claims dataset also includes provider and member-specific details such as provider specialty, geo-location of patients and providers, paid amounts, etc. To make the models generalizable and not biased towards specific providers, specialties, or geographies, we avoid using these features in our models. Moreover, some features such as provider specialty are often self-reported and could be out of date or misrepresented.

To ensure consistency across claims submitted by healthcare providers, standardized sets of diagnosis and procedure codes are used. An ICD-10 code used to report a specific diagnosis consists of seven alphanumeric characters. The first three characters represent the general diagnosis category. They are followed by a decimal point and four additional characters that specify details of the diagnosis. In our data all claims have a primary diagnosis code and optionally up to two additional diagnosis codes. An example of an ICD-10 code is as follows:

- S99.919A:
  - ◦ S99 (general category code): Injury, poisoning, and certain other consequences of external causes
  - ◦ S99.919A (full ICD-10 code): Unspecified injury unspecified ankle initial encounter

To report a specific procedure, a CPT or HCPCS code is used. These codes are developed and maintained by the American Medical Association and are assigned to specific medical actions. A procedure code generally is a five-digit numeric code, but some contain a letter. An example of a CPT code is as follows:

- 73600:
  - ◦ Category: Radiological Services (Category I CPT)
  - ◦ Procedure Description: X-ray ankle 2.0 views

As an example, consider a claim submitted for a thirty-five-year-old woman who goes to a healthcare provider with a broken ankle. The general claim information along with features that we consider for our machine learning models are shown in Table 1. Claims typically consist of multiple claim lines where each claim line contains only one procedure, and multiple procedures are often performed for a single claim.

**Table 1:** Generalized claim information for a patient with a broken ankle

Claim Number	ICD-10 Code	Diagnosis	CPT Code	Procedure	Age	Gender
1	S99.919A	Ankle Injury	73600	X-ray ankle	35	F
1	S99.919A	Ankle Injury	73615	Review X-ray to determine if ankle is broken	35	F
1	M84.371A	Broken Ankle	L2108	Set broken ankle in a cast	35	F
1	M84.371A	Broken Ankle	E0112	Prescribe underarm crutches	35	F

Input features to the machine learning models are one- or multi-hot encoded. Age is bucketed into five categories: under 18, 18 to 38, 39 to 59, 60 to 80, and 81 and older, and the five buckets are one-hot encoded. Gender consists of a one-hot encoded vector with three categories – male, female, and other. Since a claim can have more than one CPT and ICD-10 codes, these are multi-hot encoded. Lengths of these multi-hot encoded feature representations correspond to the total number of distinct ICD-10 and CPT codes found in the dataset. To reduce sparsity of the feature space, we only use the general category of the ICD-10 codes. Since the models predict claims that contain outlier procedures, we eliminate CPT codes that occur in less than one-hundred claims within the entire dataset to ensure that the models do not flag procedures as outliers based solely on their rarity in the dataset.

### Modeling approaches

An unsupervised machine learning model trained on claims data containing the aforementioned features learns specific combinations of procedure codes that are typically associated with the other features in the data. An outlier procedure code is one that a model identifies as not belonging with the other combinations of procedures, diagnoses, and demographic information. For model selection we evaluated a density-based clustering approach and multiple variations of autoencoder models that have been shown to work well for anomaly detection with sparse feature representations [23].

- A) Density-based spatial clustering of applications with noise (DBSCAN)

DBSCAN [24], which is commonly used for anomaly detection, including detecting medical fraud and predicting medical costs [25–27], was used as a baseline method. It works by clustering nearest neighbor data, making it possible to identify anomalies that are not associated with any clusters. Compared to other clustering approaches such as k-Nearest Neighbors, DBSCAN is less suspectable to noise, can derive the number of clusters automatically, and find arbitrarily shaped clusters. Hyper-parameter choices of the trained model are shown in the Supplementary File.

- B) Autoencoder

We experimented with autoencoders [28–30], which have been used in a wide variety of applications ranging from producing reduced representations of nonlinear, multivariant data [31, 32], to anomaly detection in various domains [33–37], including fraud detection in Medicare claims [9, 38].

An autoencoder consists of an encoder, a compressed latent space or “bottleneck” layer, and a decoder. The encoder and the decoder typically comprise of one or more fully connected layers. The encoder layers often consist of progressively fewer number of nodes that learn how to compress the input feature representations into a smaller latent space in a way that retains important information about the features. The decoder reverses this compression process. An autoencoder trained on normal data learns to retain only the most relevant features of the data to be able to reconstruct the input. An anomalous input to this trained autoencoder typically results in a large difference between the input and the reconstructed output resulting in a large reconstruction error. For our data, this allows us to detect specific procedure codes within medical claims that do not belong with the other procedure codes, diagnosis codes and demographic data.

**Fig. 1:**
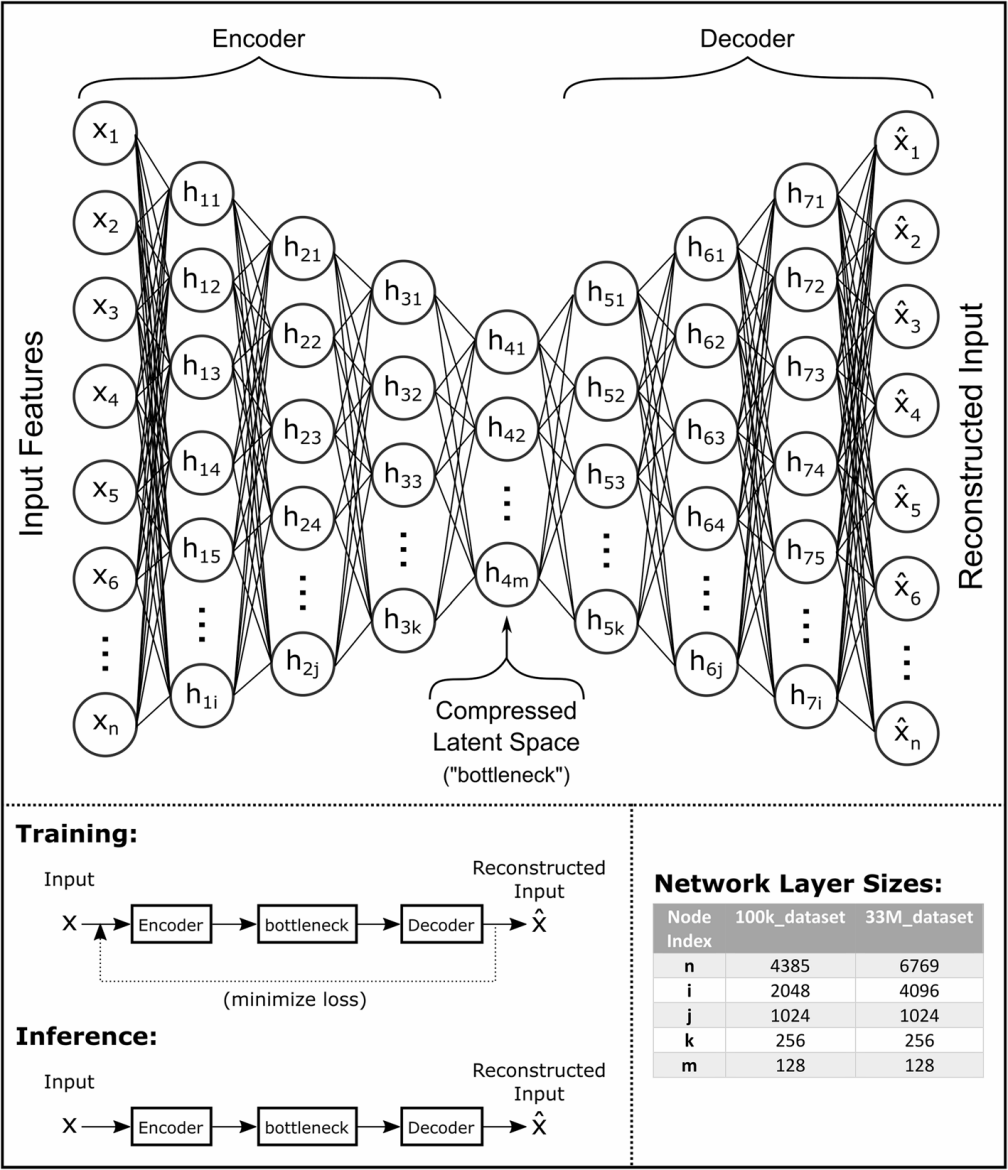
A diagram of the autoencoder model used in this study

The neural network structure for the deep autoencoder containing seven hidden layers used in this study is shown in Fig. 1. For the 100k_claims dataset, the number of nodes of the input and output layers (corresponding to the input feature representation dimensionality) is 4835, and the dimensionality of the bottleneck layer is 128. For the 33M_claims dataset, the number of nodes of the input and output layers increase to 6769 due to increased dimensionality of the ICD and CPT feature encodings, but the latent space and model structure otherwise remains the same. Each encoder layer and all but the last decoder layer consists of a linear transform followed by a rectified linear unit (ReLU) activation function. The output layer of the decoder consists of a linear transformation followed by a sigmoid activation function. Since the input feature vector consists of just zeros and ones, the decoder’s sigmoid output ensures that its reconstructed output data ranges from zero to one. Details of hyper-parameter tuning and final hyper-parameter choices are shown in the Supplementary File.

#### Feature-weighted loss function

Training an autoencoder on a large sparse input feature representation is challenging. During model training, the sparsity of the input features makes it easy for the model to learn to simply predict all zeros since this consistently produces a small loss or reconstruction error. In order to address this problem, we propose to use a custom weighted loss function. By weighting the ones more heavily than zeros, the weighted loss function penalizes the model for predicting zeros in the reconstructed output vector where there should be ones (based on input feature representations).

Since the inputs and outputs of the model are one-hot encoded (binary) we selected a binary cross entropy (BCE) loss function for optimizing the model during training. A weighed binary cross entropy (wBCE) loss function typically applies weights based on rarity of individual classes. For a detailed comparison of BCE and wBCE loss functions, refer to the paper by Ho and Wookey [39]. The loss function we use in this study is a variation of the wBCE loss. Specifically, we are using a feature-weighted BCE (fwBCE) loss function that applies weights based on the ones and zeros for each observed output vector.

The standard BCE loss function is given by the following equation:

$$\mathrm{BCE}\;\mathrm{Loss}=-\frac1{\mathrm N}\sum_{\mathrm i=0}^{\mathrm N}\sum_{\mathrm j=0}^{\mathrm M}\left[{\mathrm y}_{\mathrm{ij}}\log{\widehat{\mathrm y}}_{\mathrm{ij}}+\left(1-{\mathrm y}_{\mathrm{ij}}\right)\log\left(1-{\widehat{\mathrm y}}_{\mathrm{ij}}\right)\right]$$

where:

M is the feature vector length

N is the batch size

${\mathrm{y}}_{\mathrm{ij}}$ is the target (0 or 1 from the feature vector)

${\widehat{\mathrm{y}}}_{\mathrm{ij}}$ is the predicted probability of class 1

(1 – ${\mathrm{y}}_{\mathrm{ij}}$) is the predicted probability of class 0

The fwBCE loss function used in this study is the same as in the above equation, except for an added weighting term (w) as in the following equation:

$${\text{fwBCE Loss}}=-\frac1{\mathrm N}\sum_{\mathrm i=0}^{\mathrm N}\overset{\mathrm M}{\underset{\mathrm j=0}{\sum\;}}\left[{(\mathrm y}_{\mathrm{ij}}\log{\widehat{\mathrm y}}_{\mathrm{ij}})+{\mathrm w}\left(\left(1-{\mathrm y}_{\mathrm{ij}}\right)\log\left(1-{\widehat{\mathrm y}}_{\mathrm{ij}}\right)\right)\right]$$

Here w is the weight applied to the values associated with the feature vector (${\mathrm{y}}_{\mathrm{ij}}$) that are zero. No weighting is applied to values associated with the feature vector (${\mathrm{y}}_{\mathrm{ij}}$) that are one. In practice, w is between 0 and 1. The closer w is to 0, the less influence false positives have on the total loss.

### Model evaluation

To evaluate the unsupervised models, we developed two test datasets: an out-of-sample outlier dataset and a manually annotated outlier dataset. Both datasets are designed to meet the following criteria:

- None of the claims in the test dataset are contained in the training dataset.
- The features of the test dataset are encoded identically to the training dataset resulting in feature vectors that are of the same length as the training dataset.
- The outliers represent the minority class in an imbalanced dataset – as typically seen in real-world datasets.

#### Out-of-sample outlier test dataset generation

These test datasets were generated by sampling claims from a larger claims dataset and introducing outlier procedure codes to a small fraction of the claims. Claims with the outlier procedures (i.e. outlier claims) have one or two CPT codes added that do not appear in our training dataset, or the subset of the test dataset containing claims without outlier procedures (i.e. normal claims). Half of the outlier claims have one out-of-sample CPT added, and the other half has two out-of-sample CPTs added. Since the out-of-sample CPTs change based on training data for the models, two out-of-sample test datasets were generated, one for the 100k_claims dataset and another for the 33M_claims dataset. Both test datasets comprised of 10,000 samples with outliers comprising of 27% and 20% of the samples, respectively. The out-of-sample test dataset is used to evaluate how well the trained models detect outlier CPT codes that were absent from the training set.

#### Manually annotated outlier dataset generation

A second test dataset manually annotated by a FWA SMS is used for further evaluation of the trained models. This dataset consists of 160 claims that are annotated to denote whether each claim contains one or more CPT codes that indicate overutilization. 6 claims were unlabeled resulting in a final set of 154 annotated claims. A claim containing an outlier CPT code is considered an outlier. Only 30% of the manually annotated claims are labeled as outliers.

The demographic variable distributions in the manually annotated and the out-of-sample test datasets were proportionate to the corresponding distributions in the 33M_claims dataset.

Model performance was measured on the two test datasets using standard classification metrics: precision, recall, and F1-score. The targets in the test datasets are zero for a claim with no outlier CPT codes and one for claims with at least one outlier CPT code. Since we are interested in model performance for detecting outliers, the outlier class is assumed to be the positive class in this study.

Precision is a measure of how many positive class predictions are correct. It is a good metric to use when the cost of false positive is high. In this study, false positives mean CPT codes are incorrectly identified as outliers, which results in healthcare regulators spending more time and resources identifying actual cases of procedure overutilization. Precision is calculated as follows:

$$\mathrm{Precision}=\frac{True\;Positive}{True\;Positive\;+\;False\;Positive}=\frac{True\;Positive}{Total\;\;Predicted\;Positive}$$

Recall, which is also referred to as sensitivity, is a measure of how many positive classes the model correctly predicts versus all the positive cases in the data. It is a good metric to use when there is a high cost associated with false negatives. For this study, a false negative means an outlier procedure that could represent overutilization is not detected. Since this model was designed for application in post-payment overutilization detection where a FWA SMS or analyst reviews the model outputs, a higher recall was desirable. Recall is calculated as follows:

$$\mathrm{Recall}=\frac{True\;Positive}{True\;Positive\;+\;False\;Negative}=\frac{True\;Positive}{Total\;Actual\;Positive}$$

F1-score is the harmonic mean of precision and recall, and it is a useful metric when seeking a balance between both metrics, particularly when there is imbalanced data with a large negative class. F1-score is calculated as follows:

$$\mathrm{F}1-\mathrm{score }=2 \times \left(\frac{\mathrm{Pr}ecision \times Recall}{\mathrm{Pr}ecision + Recall}\right)$$

The models infer a probability score for each CPT code in a claim. A predicted value near one indicates the CPT code is not an outlier, and a predicted value near zero indicates the CPT code is an outlier. To compare inference results with targets in the test datasets, the individual CPT scores are aggregated to generate a single score between zero and one for each claim such that a score near zero indicates no outlier is present and a score near one indicates at least one CPT in the claim is an outlier.

Threshold values between 0.0 and 1.0 in increments of 0.05 were applied to the aggregate claim scores. Any aggregate claim score less than the threshold was assigned a value of 0.0, and any score greater than or equal to the threshold was assigned a value of 1.0. For each threshold value, these assigned claim values were then used to calculate precision, recall, and F1-score based on target values for each claim in the test datasets. The performance metrics at the threshold that produced the maximum F1-score were used in model performance evaluations.

## Results

**Fig. 2:**
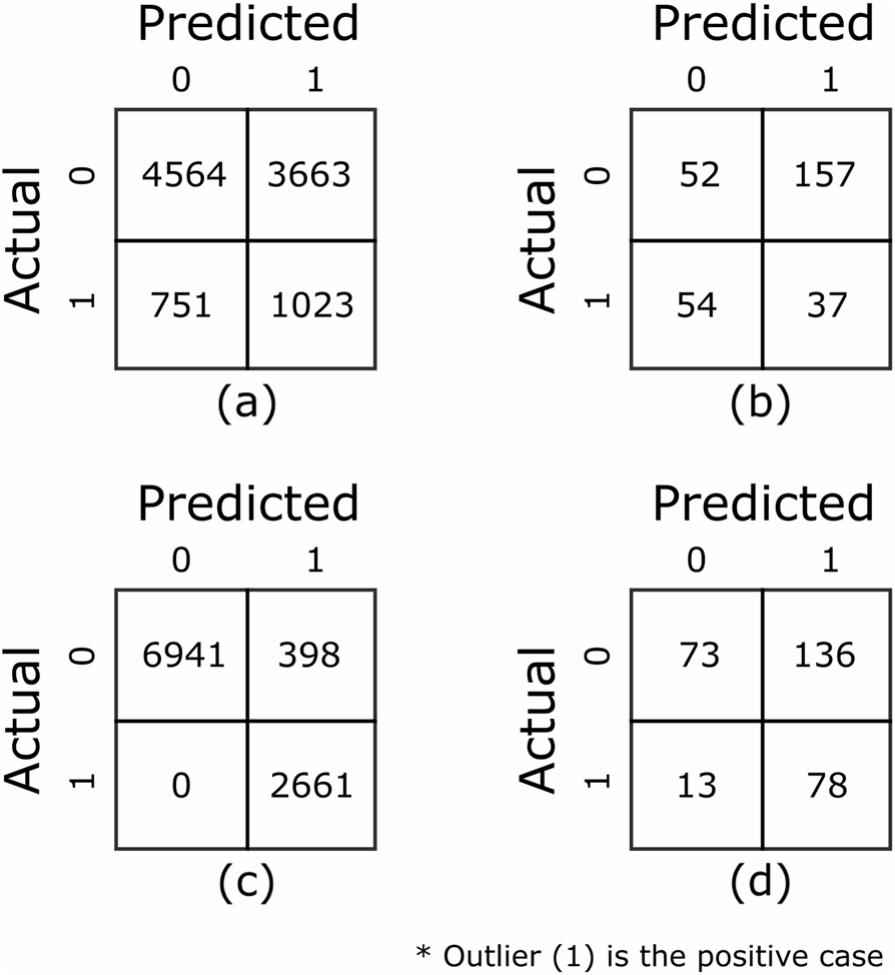
Confusion matrices on test datasets for models trained on 100k_claims dataset showing (**a**) DBSCAN out-of-sample, (**b**) DBSCAN manually annotated, (**c**) Autoencoder out-of-sample, and (**d**) Autoencoder manually annotated

Performance metrics of the DBSCAN baseline and the autoencoder models trained on the 100k_claims dataset is summarized in Table 2. The confusion matrices for these models are plotted in Fig. 2. The synthesized out-of-sample test data contains “outlier” CPT codes that have never been seen by the models during tuning and training. The DBSCAN model produced poor performance metrics on this test dataset with an F1-score of 0.32, recall of 0.58, and precision of 0.22. In contrast, the autoencoder model performed well producing an F1-score of 0.93, recall of 1.0, and precision of 0.87. In other words, the autoencoder model produced no false negatives and very few false positives.

**Table 2:** Performance metrics of models trained on 100k_claims dataset

**Test Datasets**	**Metrics**	**DBSCAN**	**Autoencoder**
Out-of-sample	Precision	0.22	**0.87**
	Recall	0.58	**1.0**
	F1	0.32	**0.93**
Manually annotated	Precision	0.19	**0.36**
	Recall	0.41	**0.86**
	F1	0.26	**0.51**

Unlike the out-of-sample test data, the manually annotated test data contains CPT codes the models saw during training, making it more difficult to identify specific outlier CPTs within a claim. Again, the DBSCAN model performed poorly with an F1-score of 0.26, recall of 0.41, and precision of 0.19. The autoencoder model performed much better with an F1-score of 0.51, recall of 0.86, and precision of 0.36. As shown in the confusion matrices on Fig. 2, while the autoencoder model minimized false negatives it produced a significant number of false positives. McNemar's statistical hypothesis test [40, 41] shows that the performance improvements with the autoencoder models compared with the baseline DBSCAN models are statistically significant with *p*-values < 0.001.

As stated earlier, model performance on the 100k_claims dataset was used for benchmarking and model selection for production. Based on the above results we trained an autoencoder model on the 33M_claims dataset as our final model for production.

**Fig. 3:**
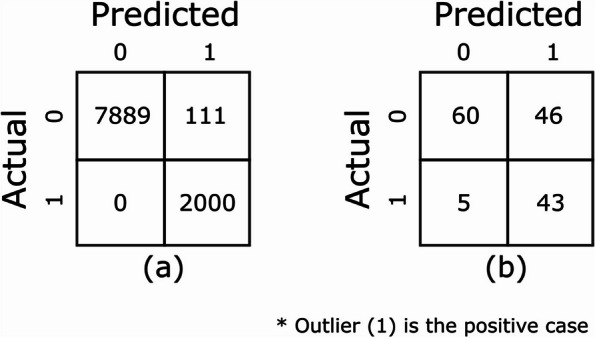
Confusion matrices of models trained on 33M_claims dataset for (**a**) out-of-sample and (**b**) manually annotated test datasets

The performance metrics for the autoencoder model on the out-of-sample and manually annotated test datasets are shown in Table 3. The confusion matrices for these models are shown in Fig. 3. The model trained on 33M_claims dataset outperformed the one trained on 100k_claims dataset for both the test datasets. For the out-of-sample dataset the F1 score was 0.97 with a precision of 0.95. On the manually annotated dataset the F1 score improved to 0.63 vs 0.51, precision increased to 0.48 vs 0.36 and recall improved to 0.90 vs 0.86.

**Table 3:** Performance metrics of autoencoder model trained on 33M_claims dataset

**Test Datasets**	**Metrics**	**Scores**
Out-of-sample	Precision	0.95
	Recall	1.0
	F1	0.97
Manually annotated	Precision	0.48
	Recall	0.90
	F1	0.63

## Discussion

Table 4 shows examples of procedures that were identified to be overutilized by the autoencoder model, given the diagnosis conditions and demographics of the patients. For claim #1, while an outpatient visit and one unit of removal of growth of the trunk arms or legs for seborrheic keratosis may be warranted, the presence of multiple procedures of different units for one instance of the growth is questionable. For claim #2, for a patient with Dorsalgia, the presence of procedure codes 97811 and 97814 appears to be overutilized in the presence of procedures codes 99213 and 97813 with unclear justification of their usage given the diagnosis code.

**Table 4:** Examples of claims where autoencoder model predictions were consistent with manual annotations. Italicized cells denote model predictions for overutilization

Claim #	ICD-10 Code	Diagnosis	CPT Code	Procedure	Age	Gender	FWA SMS comments
1	L82	Seborrheic keratosis	*11401*	*Removal of growth (0.6 to 1.0 centimeters) of the trunk arms or legs*	65	F	CPT 11401 and 11400 are questionable if there is only one growth. One removal - units and detailed diagnosis would need further review
			99212	Established patient outpatient visit total time 10-19 minutes			
			*11400*	*Removal of growth (0.5 centimeters or less) of the trunk arms or legs*			
2	M54	Dorsalgia	99213	Established patient outpatient visit total time 20-29 minutes	36	F	CPT 97811 and 97814 appears to be overutilized in the presence of the other CPT codes
			97813	Acupuncture 1 or more needles with electrical stimulation first 15 minutes			
			*97814*	*Acupuncture 1 or more needles with electrical stimulation and re-insertion of needles*			
			*97811*	*Acupuncture 1 or more needles*			

In our error analysis of the model outputs, we found rare procedure combinations were often incorrectly flagged by the model (Table 5). For example, in claim #3 while the model identified procedure code 29806 to be a potential overutilization, an incision may be performed in case a surgical procedure was needed to treat the dislocation of the shoulder. Similarly for claim #4, while the procedures 97110 and 97140 may not have been commonly seen either together or in combination of the diagnosis codes, these are not deemed to be overutilized by our annotator.

**Table 5:** Examples of claims where autoencoder model predictions with inconsistent with manual annotations. Italicized cells denote model predictions for overutilization

Claim #	ICD-10 Code	Diagnosis	CPT Code	Procedure	Age	Gender	FWA SMS comments
3	S43	Dislocation sprain and strain of joints and ligaments of shoulder girdle	*29806*	*Incision of shoulder joint capsule using an endoscope*	27	M	An incision would need to be made if surgical procedure was performed. Units would need to be taken into consideration.
3	M89	Other disorders of bone	L3670	Shoulder orthosis acromio/clavicular (canvas and webbing type) prefabricated off-the-shelf			
4	M54	Dorsalgia	*97110*	*Therapeutic exercise to develop strength endurance range of motion and flexibility each 15 minutes*	31	F	Standard practice to bill 97110 and 97140 with diagnosis for same date of service
			*97140*	*Established patient outpatient visit total time 10-19 minutes*			

We make various observations in our analysis of model performance on the out-of-sample and manually annotated test datasets. Compared to the idealized set up of the out-of-sample outlier test data, the manually annotated data provides a more challenging evaluation of our models. The large number of false positives identified by the models indicate that the 100k_claims data was likely not large enough for the models to learn all combinations of procedure codes that do not belong with specific diagnosis codes and demographic information. In addition, the manually annotated outlier test dataset contains CPT codes that are also present in the normal claims in the training data, making it more difficult for the model to accurately identify which CPT codes are outliers. Prior research [42] shows that while synthetic test data may provide a controlled environment for evaluation of outlier detection methods, they may not necessarily reflect the complexity and variability in real-world data. Therefore, our results provide a more realistic assessment of the machine learning modeling approaches across diverse datasets.

As demonstrated by the results, the autoencoder model significantly outperforms a baseline density-based clustering algorithm. Given the highly imbalanced nature of the dataset and sparsity of feature representations, the feature-weighted BCE loss function (fwBCE) played a key role in training our model. Tuning the weighting factor for the loss function was essential to creating a model that accurately reproduces the input feature vectors. With little or no weighting, the predicted output vectors essentially contain only zeros for all variations of input feature vectors. This occurs because the model learns that always predicting zeros consistently produces low loss given how few ones exist in the input vectors. In contrast, too large a weighting penalizes the model too much resulting in it predicting all ones regardless of input feature vector values. The weighting factor for the fwBCE loss function influences how the reconstructed output vectors accurately reproduce the zeros and the sparse ones in the input feature vector.

As expected, the autoencoder model trained on 33M_claims dataset outperforms the one trained on 100k_claims dataset, likely due to the availability of substantially more information for the model to learn relationships between various procedure codes, diagnosis codes, and demographic information. This model shows better performance with improved identification of true positives and better elimination of false negatives.

Our study has certain limitations. We did not consider other claim features such as healthcare provider information or billing amount which may influence prioritization of cases by fraud investigators. While we explored a deep autoencoder and various hyper-parameters to tune it, we did not explore variations of the standard autoencoder architecture, such as a variational autoencoder, or adding regularization to improve model performance. Since sparse feature representations make the autoencoder model challenging to train, in future we would like to explore whether word or graph embedding of the diagnosis and procedure codes can improve model training and tuning. We did not consider a bias and fairness study of the demographic variables in this study and would like to explore that in future.

## Conclusion

In this study, we demonstrated that unsupervised machine learning models can be used for detecting procedure code overutilization in healthcare claims. Specifically, we showed that an autoencoder can be tuned and trained to efficiently detect procedure code outliers in millions of claims. While this model may be used for automated pre-payment screening of claims, we propose its use as an automated tool to flag procedure codes that do not belong with other procedure codes or with diagnosis codes and demographic data in a healthcare claim, to be eventually verified by a human reviewer. This produces a significantly smaller set of suspicious claims that healthcare fraud specialists and investigators need to manually review in detail as part of identifying specific cases of procedure overutilization. Even a small lift in the percentage of claims identified as containing procedure overutilization means that the models described in this study could help recover millions of additional dollars that would not be possible with a fully manual process.

As indicated by an F1-score of 0.97 on the out-of-sample test dataset, the autoencoder model trained on the 33M_claims dataset can detect overutilization with a low false positive and no false negatives when certain procedures are extremely rare in combination of other procedures, diagnosis or demographics. However, on the manually annotated test dataset we notice that the model has lower F1-score of 0.63 which can be attributed to a higher number of false positives. We speculate that the discrepancy between the model performance on the out-of-sample and the manually annotated test datasets could be due to the model identifying certain procedures as outliers when it has not seen those in combination with other procedures, diagnosis and demographics, and those procedures while being very uncommon are billed appropriately according to the FWA SMS. Future work on improving model performance further will focus on improving the precision or reducing the false positives.

### Supplementary Information

**Additional file 1.**

## Data Availability

The raw datasets analyzed during the current study are not publicly available in full due to licensing and contractual restrictions, but synthetic sample dataset is available from the corresponding author on reasonable request.

## Abbreviations

AE: Autoencoder
AI: Artificial Intelligence
BCE: Binary Cross Entropy
CPT: Current Procedural Terminology
DBSCAN: Density-Based Spatial Clustering of Applications with Noise
FWA: Fraud, Waste, and Abuse
fwBCE: Feature-Weighted Binary Cross Entropy
HCPCS: Healthcare Common Procedure Coding System
ICD-10: International Classification of Diseases (revision 10)
NHCAA: National Health Care Anti-Fraud Association
NPI: National Provider Identifier
ReLU: Rectified Linear Unit
SMS: Subject Matter Specialist
SURS: Surveillance Utilization Review System
wBCE: Weighted Binary Cross Entropy
