# Supplementary material for: Procedure code overutilization detection from healthcare claims using unsupervised deep learning methods
Source: BMC Med Inform Decis Mak. 2023 Sep 28;23:196. doi: 10.1186/s12911-023-02268-3 (PMC10536726; doi:10.1186/s12911-023-02268-3)
Supplement: Supplementary file 1 — Additional file 1. [file 12911_2023_2268_MOESM1_ESM.docx]

## Pseudocode of Overutilization Detection Algorithm

Algorithm S1: Autoencoder Training and Inference

Code for Overutilization Detection

**Input**: sparse feature vector ($Xn$) consisting of encoded
CPT Codes, ICD codes, and demographic data
of length n (where n = 4385 or 6769)

**Key Hyperparameters**: learning rate ($l$) and
BCE loss weighting factor ($w$)

**Output**: reconstructed input ($\hat{Xn}$)

Instantiate autoencoder in training mode as $net$

Select hyperparameters ($l$ and $w$)

for N epochs do
for batch size j do

batch_loss = $net.fit(Xnj)$
Add batch loss to epoch_loss ($L(l,w)$)

Minimize $L(l,w)$

Repeat epoch training loop to identify optimal
hyperparameters ($l$ and $w$)

Save autoencoder parameters trained with
optimal hyperparameters ($\hat{net}$)

Use $\hat{net}$ in inference mode to generate $\hat{Xn}$ from $Xn$

Compare $\hat{Xn}$ and $Xn$ to identify
procedure code (CPT) outliers

## Model Hyper-parameter Tuning

DBSCAN: The model was optimized by selecting the *eps* parameter that produced the maximum number of clusters with the *minPts* parameter equal to 15.

Autoencoder: Hyper-parameter tuning of the autoencoder model is performed to minimize loss using a Bayesian search mechanism. The hyperparameters that were optimized are summarized in Table S1.

Table S1: Hyperparameters Optimized During Model Tuning

| **Category** | **Hyperparameters Tested** |
| --- | --- |
| Optimization Function | Adam and Stochastic Gradient Decent |
| Activation Function | ReLU and Leaky-ReLU |
| fwBCE Loss Weighting Factor | Continuous Values (from 0.0005 to 0.01) |
| Epochs | Discrete Values (from 10 to 250) |
| Learning Rate | Continuous Values (from 0.0001 to 0.01) |
| Batch Size | Discrete Values in Multiples of 2 (from 128 to 4096) |
| Dropout Factor | Continuous Values (from 0.1 to 0.4) |

As part of determining the optimal model, the above hyperparameters were tuned for the following eleven variations of model structures as shown in Table S2.

Table S2: AE Model Structures Tested

| Number of Encoder Layers | Number of Decoder Layers | Bottleneck Size |
| --- | --- | --- |
| 3 | 3 | 128 |
| 3 | 3 | 256 |
| 4 | 4 | 64 |
| 4 | 4 | 128 |
| 4 | 4 | 256 |
| 4 | 4 | 512 |
| 5 | 5 | 64 |
| 5 | 5 | 128 |
| 5 | 5 | 256 |
| 6 | 6 | 64 |
| 6 | 6 | 128 |

For each model structure, hyperparameter this tuning process occurred in multiple steps. The first tuning step used the full range of hyperparameters in [Table S1.](#T3) For the second tuning step, a new, narrower range of hyperparameters were defined around the optimal parameters from the first tuning step, and the full hyperparameter tuning process was repeated with these new hyperparameter ranges. Often a third hyperparameter tuning step was done with a narrower range of hyperparameters based on results from the second tuning step. This iterative approach allowed us to determine optimal hyperparameters with less time and resources than doing it in just one step using the full range of hyperparameters.

Once the optimal hyperparameters were determined for the eleven model structures, each model was trained using its optimal hyperparameters and then saved. The saved models were used to perform inference with the test and validation data as part of selecting the optimal model.

For the autoencoder model trained on the 100k_claims dataset, the optimal model structure consists of four layers for the encoder and decoder with a bottleneck size of 128. The optimal hyper-parameters for this model are summarized in Table S3.

Table S3: Optimal hyper-parameters for the autoencoder model trained on 100k_claims dataset

| **Category** | **Optimal Hyperparameter** |
| --- | --- |
| Optimization Function | Adam |
| Activation Function | ReLU |
| fwBCE Loss Weighting Factor | 0.003 |
| Epochs | 150 |
| Learning Rate | 0.000426 |
| Batch Size | 512 |
| Dropout Factor | 0.11 |

For the autoencoder model trained on the 33M_claims dataset, the optimal model structure consists of four layers for the encoder and decoder with a bottleneck size of 128. The optimal hyper-parameters for this model are summarized in Table S4.

Table S4: Optimal hyper-parameters for the autoencoder model trained on 33M_claims dataset

| **Category** | **Optimal Hyperparameter** |
| --- | --- |
| Optimization Function | Adam |
| Activation Function | ReLU |
| fwBCE Loss Weighting Factor | 0.0494 |
| Epochs | 25 |
| Learning Rate | 0.00016 |
| Batch Size | 512 |
| Dropout Factor | 0.13 |

The optimal number of epochs (25) was selected based on the loss plot shown in Figure S1.

Figure S1: Loss plot for the autoencoder model trained on 33M_claims dataset
